# Missense Mutations in LRP5 Associated with High Bone Mass Protect the Mouse Skeleton from Disuse- and Ovariectomy-Induced Osteopenia

**DOI:** 10.1371/journal.pone.0140775

**Published:** 2015-11-10

**Authors:** Paul J. Niziolek, Whitney Bullock, Matthew L. Warman, Alexander G. Robling

**Affiliations:** 1 Department of Anatomy & Cell Biology, Indiana University School of Medicine, Indianapolis, Indiana, United States of America; 2 Weldon School of Biomedical Engineering, Purdue University, West Lafayette, Indiana, United States of America; 3 Department of Orthopaedic Surgery, Children’s Hospital, Boston, Massachusetts, United States of America; 4 Howard Hughes Medical Institute, Department of Genetics, Harvard Medical School, Massachusetts, United States of America; 5 Department of Biomedical Engineering, Indiana University–Purdue University at Indianapolis (IUPUI), Indianapolis, Indiana, United States of America; 6 Richard L. Roudebush VA Medical Center, Indianapolis, Indiana, United States of America; Oklahoma State University, UNITED STATES

## Abstract

The low density lipoprotein receptor-related protein-5 (LRP5), a co-receptor in the Wnt signaling pathway, modulates bone mass in humans and in mice. Lrp5 knock-out mice have severely impaired responsiveness to mechanical stimulation whereas Lrp5 gain-of-function knock-in and transgenic mice have enhanced responsiveness to mechanical stimulation. Those observations highlight the importance of Lrp5 protein in bone cell mechanotransduction. It is unclear if and how high bone mass-causing (HBM) point mutations in Lrp5 alter the bone-wasting effects of mechanical disuse. To address this issue we explored the skeletal effects of mechanical disuse using two models, tail suspension and Botulinum toxin-induced muscle paralysis, in two different Lrp5 HBM knock-in mouse models. A separate experiment employing estrogen withdrawal-induced bone loss by ovariectomy was also conducted as a control. Both disuse stimuli induced significant bone loss in WT mice, but Lrp5 A214V and G171V were partially or fully protected from the bone loss that normally results from disuse. Trabecular bone parameters among HBM mice were significantly affected by disuse in both models, but these data are consistent with DEXA data showing a failure to continue growing in HBM mice, rather than a loss of pre-existing bone. Ovariectomy in Lrp5 HBM mice resulted in similar protection from catabolism as was observed for the disuse experiments. In conclusion, the Lrp5 HBM alleles offer significant protection from the resorptive effects of disuse and from estrogen withdrawal, and consequently, present a potential mechanism to mimic with pharmaceutical intervention to protect against various bone-wasting stimuli.

## Introduction

Osteoporosis is a low-bone-mass disease resulting from impaired bone modeling or imbalanced bone remodeling, in which resorption outpaces apposition, resulting in bones that are fragile and prone to fracture [1]. Osteoporotic fractures are associated with chronic pain, decreased quality of life, and large financial cost. Pathological bone loss also occurs in patients that are immobilized due to bed rest [2, 3], muscle paralysis [4, 5], or have decreased mechanical loading to the bone [6]. In paraplegic patients, the disuse-induced bone loss leads to increasing risk of fractures in the lower limbs with time [4].

The Wnt signaling cascade, which was initially characterized as a pathway involved in neural tube development, limb patterning, planar cell polarity, axon guidance, and cancer, has more recently emerged as a key pathway mediating bone homeostasis [7–12]. Consequently, there has been great interest in understanding whether and how Wnt signaling perturbations might be involved in the development of osteoporosis [13, 14]. Perhaps more importantly, this pathway is currently being probed to elucidate whether pharmacologic manipulation of Wnt signaling in bone might lead to additional and potentially more effective treatments for osteoporosis [15]. The interest in Wnt’s role in osteoporosis biology has extended to disuse osteoporosis [16, 17], and work in this area has yielded promising results [18–20].

Wnt proteins interact with the surface receptors low density lipoprotein receptor-related proteins 5 or 6 (LRP5, LRP6) and a family of co-receptors—the Frizzled proteins. Mutations in LRP5 can lead to vastly different skeletal effects in humans, ranging from severely low bone mass [14] to a very high bone mass (HBM) phenotype [21, 22]. LRP5 HBM-causing mutations are of particular therapeutic interest as they may hold clues for effective treatments for osteoporosis and disuse-induced bone loss. The molecular mechanism by which these mutations lead to high bone mass is becoming elucidated, and likely involves resistance to soluble LRP5/6 antagonists, including sclerostin, Dkk1, and Wise [10, 23–30].

We have recently found that mice harboring the G171V or A214V HBM mutations within the Lrp5 coding sequence are resistant to the osteopenic effects of excessive sclerostin exposure, but mice with the G171V mutation exhibited greater resistance to excessive Dkk1 exposure that did mice with the A214V mutation [29]. Furthermore, we found that mechanical loading reduces Sost expression, whereas forced overexpression of Sost prevents mechanically induced bone gain [31, 32]. Conversely, increased expression of Sost is required for tail suspension-induced bone loss [18]. In light of our previous observations that (1) the Lrp5 G171V and A214V mutations protect against Sost-induced bone loss, and (2) Sost has an important role in mechanotransduction signaling, we hypothesized that the Lrp5 G171V and A214V HBM knock-in mice would be resistant to bone loss under mechanically induced bone-wasting stimuli.

In this communication, we examine the role of Lrp5 HBM mutations in modulating the catabolic effects of disuse, using two different disuse models. First, a hindlimb suspension model was used, which suspends and unloads the hindlimbs, while allowing the forelimbs to maintain contact with the cage floor. This method prevents ground reaction forces in the hindlimbs, and has been used to model disuse-induced bone loss as seen in bedridden patients and among astronauts that experience a weightless environment.[33] Second, a muscle paralysis model was used by injecting small volumes of Botulinum toxin A (Botox) in the right hindlimb musculature to paralyze and thus underload this limb. The Botox model may be a better model for representing localized disuse osteoporosis, as seen in patients with muscular impairment or spinal cord injury [34]. Finally, we conducted an additional catabolic control experiment to probe the specificity of Lrp5 HBM mutations in regulating mechanically derived bone wasting processes, by exposing a set of mice to a non-mechanical (hormonal) catabolic stimulus. The ovariectomy (OVX) model was used to model post-menopausal bone loss associated with estrogen deficiency. Overall, these experiments allowed us to determine whether the Lrp5 HBM mutant receptors confer bone-protective effects in an environment of reduced mechanical stimulation, and whether those effects are different than those yielded by a non-mechanical bone-wasting stimulus.

## Materials and Methods

### Mice

This study was carried out in strict accordance with the recommendations in the Guide for the Care and Use of Laboratory Animals of the National Institutes of Health. The protocol was approved by the Indiana University Institutional Animal Care and Use Committee (IACUC). Generation of mice with the high-bone-mass-causing Lrp5 mutations A214V and G171V knocked-in the Lrp5 locus was described previously [35]. Briefly, two targeting constructs spanning introns 2–4 were generated, which harbored the p.G171V or p.A214V missense mutations located in exon 3. The mice were bred to homozygosity (Lrp5^+/+^, Lrp5^A214V/A214V^, or Lrp5^G171V/G171V^; henceforth deonted as WT, A214V, and G171V, respectively). The genetic background of all mice was a uniform mixture of 129S1/SvIMJ and C57Bl/6J.

### Hindlimb Suspension

Forty-eight 10 week old female mice were enrolled with 16 mice of each Lrp5 genotype (WT, A214V, G171V) and each genotype further divided into control and hindlimb-suspended mice (n = 8/group). All mice were individually housed and a tail harness was used to suspend the experimental mice as previously described.[31] Control mice were permitted unencumbered normal movement in their cages. Mice received intraperitoneal injections of tetracycline (120 mg/kg) 3 days prior to suspension and calcein (18 mg/kg) 3 weeks later. Mice were suspended for a total of 24 days prior to sacrifice.

### Botulinum toxin-induced muscular paralysis

Twenty-four 16 week old male mice were enrolled with 8 mice of each Lrp5 genotype (WT, A214V, G171V) The right hindlimb musculature (quadriceps, triceps surae, tibialis anterior, hamstrings) was injected with 20 μL of Botulinum Toxin A (Botox; Allergan Inc., Irvine, CA), while the left hindlimb musculature was injected identically with 20 μL of saline to serve as an internal control [36]. The injections were repeated one week later to ensure paralysis. Mice received injections of tetracycline (intraperitoneal 60 mg/kg, subcutaneous 60 mg/kg) the day prior to Botox administration and an intraperitoneal injection of calcein (18 mg/kg) 16 days later. Mice were sacrificed 22 days after the first Botox injection. Mice were able to access food and water without difficulty.

### Ovariectomy (OVX)

Forty-seven 10 week old female mice were enrolled with 16 mice of the WT and A214V genotype and 15 mice of the G171V genotype. Each genotype was further divided into control (sham) and ovariectomized (ovx) mice (n = 8/group, except n = 7 in G171V ovx group). Mice received intraperitoneal injections of tetracycline (120 mg/kg) the day prior to surgery and alizarin (20 mg/kg) 3 weeks later. Mice were sacrificed 27 days after surgery. For each mouse, the uterus was carefully dissected, checked for presence or absence of ovaries, and weighed to assess for atrophy due to estrogen deficiency.

### Dual energy x-ray absorptiometry (DEXA)

Whole-body *in vivo* DEXA scans were collected for each experiment on a GE Lunar PIXImus2, two days prior to the start of the experiment and again at sacrifice. Mice were anesthetized with isofluorane (2% @ 1.5 liters/min) during the procedure. Whole-body areal bone mineral density (aBMD) and bone mineral content (BMC) were calculated for the entire post-cranial skeleton among mice in the ovariectomy experiment. Femoral aBMD and BMC were measured for the hindlimb suspension and Botox experiments by adjusting the region of interest box to accommodate only the femur.

### Micro-computed tomography (μCT)

The right femur and 5^th^ lumbar vertebra were extracted at sacrifice to use in μCT analyses (Skyscan 1172). The bones were placed in 10% NBF for 2 days and then stored in 70% ethanol at 4°C. At the time of scanning, the femurs or L5 vertebrae were placed in a tube containing a thermoreversible gel (Pluronic^®^ F108) to securely immobilize the bones during the scan [37]. Acquisition settings were 60 kV, 1000x524 camera size, 0.4° step size, and a resolution of 10.04 microns/pixel. Trabecular bone was segmented from cortical bone manually using the Skyscan software package CTAn. The distal femur analysis region was 100 slices (~1.0 mm) in height and the L5 region encompassed the entire trabecular volume between growth plates.

### Dynamic Histomorphometry

Following microCT measurement, femurs were dehydrated in graded alcohols, cleared in xylene, and embedded in methylmethacrylate following standard protocols [31]. Thick sections were cut from the midshaft using a diamond wafering saw, ground down to ~30 μm, mounted on standard microscope slides, and coverslipped unstained. The femoral sections were digitally imaged on a fluorescent microscope using filter sets that provide excitation and emission for the tetracycline, calcein, and alizarin wavelengths. Digital images were imported into ImagePro Express (Media Cybernetics, Inc., Gaithersburg, MD) and the following histomorphometric measurements were recorded for the endosteal and periosteal surface: total bone perimeter (B.Pm), single label perimeter (sL.Pm), double label perimeter (dL.Pm), double label area (dL.Ar), total bone area and marrow area. The following results were calculated: mineral apposition rate (MAR = dL.Ar/dL.Pm/49 days), mineralizing surface (MS/BS = (0.5* sL.Pm + dL.Pm)/B.Pm*100), and bone formation rate (BFR/BS = MAR * MS/BS * 3.65). Tissue processing error led to the loss of two slide samples in the Botox experiment (n = 6 for the G171V mice).

### Statistical Techniques

For the tail suspension and OVX studies, pre-intervention and post-intervention DEXA measurements were compared. A paired t-test was used to determine if a difference occurred between the initial and final measurements within an Lrp5/treatment group. The percent change of a measurement from initial to final time points for each animal was calculated and the means of these percent changes were compared using student’s t-test within an Lrp5 genotype (between treatments). For microCT and histomorphometry, the values within an Lrp5 genotype were compared using student’s t-test (paired t-test for Botox mice). Statistical significance was taken at p < 0.05. Data are presented as mean ± standard devieation.

## Results

### Lrp5 G171V and A214V HBM mutations impart partial protection against tail suspension-induced bone loss

**Fig 1:**
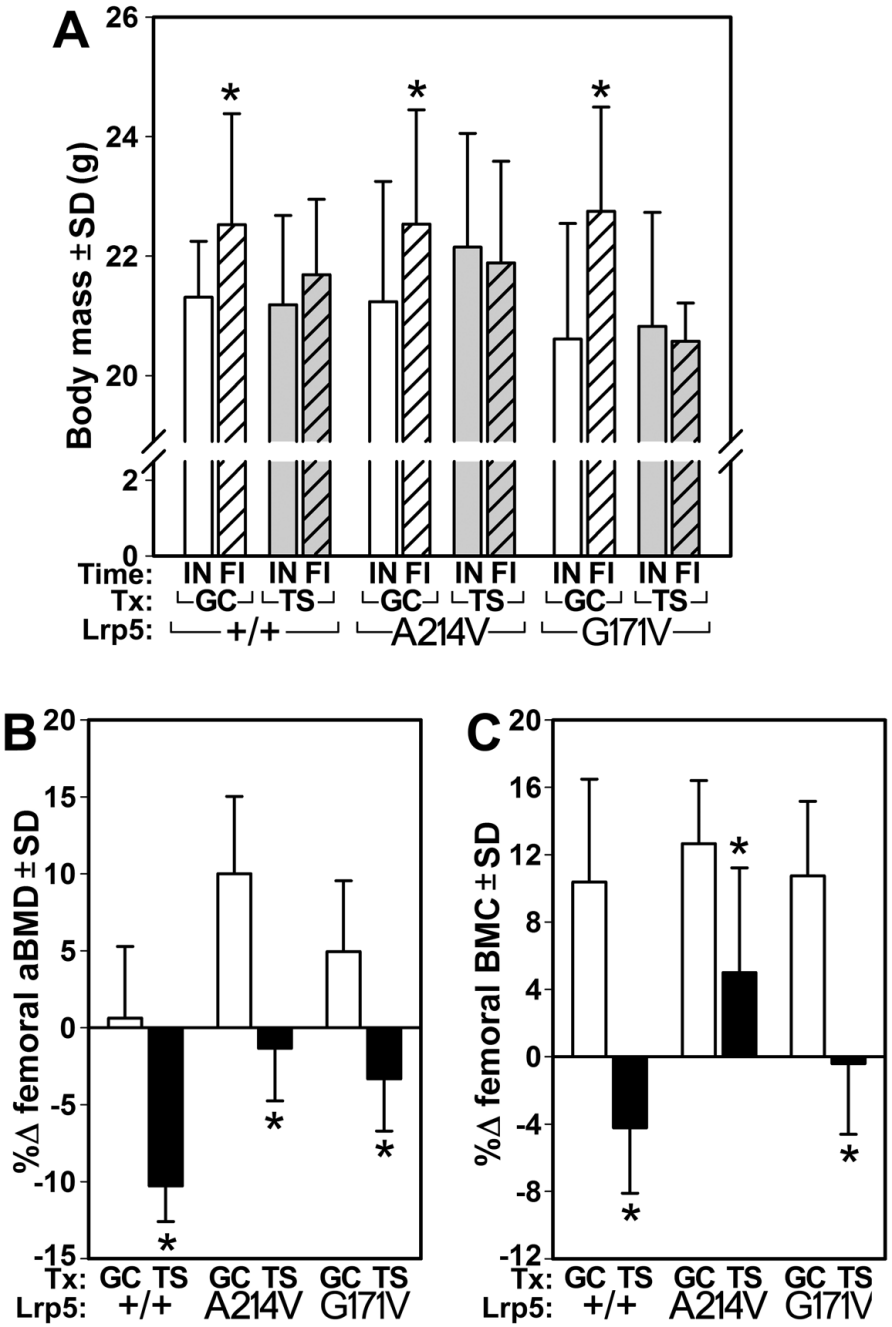
(A) Initial (IN) and final (FI) body weight in ground control (GC) and tail suspended (TS) mice. Percent change in femoral bone mineral density (B) and content (C) calculated from DEXA scans collected just prior to the start of the experiment and again at sacrifice. *p<0.05 for tail suspended mice vs genotype-matched control mice. *n* = 8/group.

To evaluate the bone-wasting effects of a fluid-shift disuse model applied to Lrp5-HBM mice, we evaluated the effects of tail suspension on bone mass, density, and structural properties in Lrp5 WT and HBM mutant mice. Regardless of genotype, all ground control mice gained body weight, whereas the tail suspended mice did not gain nor lose a significant amount of body weight (Fig 1A). WT ground control mice maintained femoral aBMD but gained 10% (p<0.01) of their initial femoral BMC over the duration of the experiment (Fig 1B and 1C). Tail suspended WT mice lost 10% femoral aBMD (p<0.001) and 4% femoral BMC (p<0.05) over the course of the experiment. As expected, HBM mutants had significantly greater aBMD (25–27%, p<0.001) and BMC (30–46%, p<0.001) compared to WT mice at the start of the experiment. Both A214V and G171V ground control mice gained a significant amount of aBMD (10% and 5%, respectively; both *p*<0.05) and BMC (13%, 11%, respectively; both *p*<0.001) over the study duration. Tail suspended A214V and G171V mice exhibited no significant loss of femoral BMC over the experimental period (change not significantly different from zero), but G171V mice lost a modest but statistically significant 3% aBMD (p<0.05).

**Fig 2:**
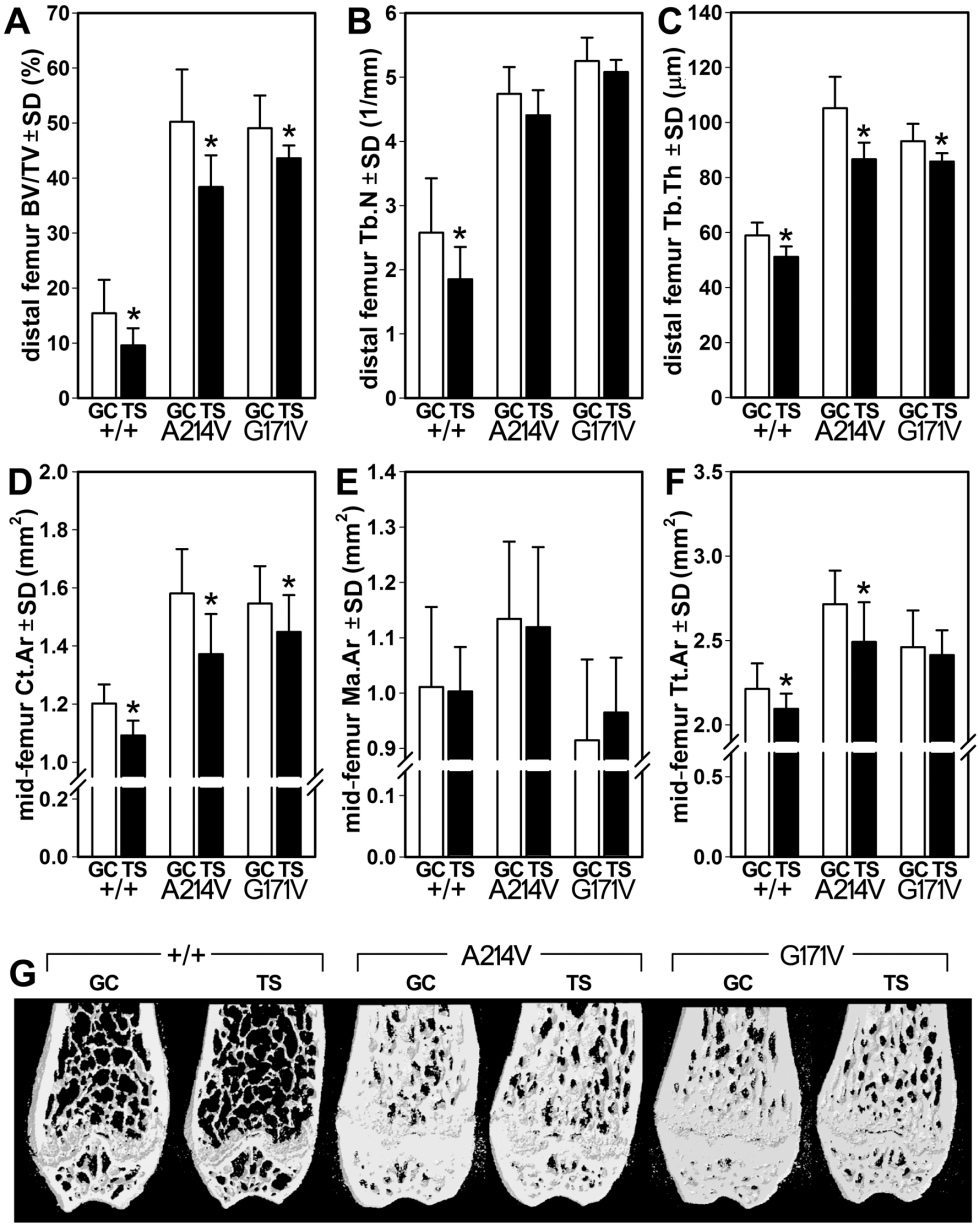
μCT measurements of cancellous and cortical bone properties in the femur of ground control (GC) and tail suspended (TS) mice. (A) Bone volume fraction, (B) trabecular number, and (C) trabecular thickness were measured in the distal femur metaphysis. (D) Cortical area, (E) medullary area, and (F) total tissue area were measured in the mid-diaphyseal femur. (F) Representative μCT reconstructions of the distal femur from GC ad TS mice within each genotype. The anterior and posterior thirds of each reconstruction have been removed digitally to reveal the metaphyseal spongiosa. *p<0.05 for tail suspended mice vs genotype-matched control mice. *n* = 8/group.

Analysis of distal femur cancellous bone by μCT indicated that tail suspension reduced BV/TV by 38% (*p*<0.05), compared to WT ground control mice (Fig 2A and 2G). Consistent with our earlier reports,[29, 38, 39] the ground control A214V and G171V mice had dramatically increased distal femur BV/TV, reaching 226% and 219% greater values, respectively (*p*<0.001) than the ground control WT control mice. Tail suspension resulted in 24% and 11% lower BV/TV (*p*< 0.05) in A214V and G171V mice, respectively, compared to their genotype-matched ground control relatives (Fig 2A). Trabecular number was significantly reduced in tail suspended WT mice (*p*<0.05) but not in tail suspended A214V or G171V mice (Fig 2B). Trabecular thickness was reduced significantly by tail suspension in all three genotypes (*p*<0.05; Fig 2C). Cancellous parameters in the fifth lumbar vertebral body (L5) followed similar trends as were observed in the femoral metaphysis (S1 Fig and S1 Table). Midshaft femur cortical area was significantly reduced by tail suspension in all three genotypes (6–13% reduction, *p*<0.05; Fig 2D), whereas medullary area was statistically unchanged by tail suspension in all three groups (Fig 2E). However, total tissue area at the midshaft was significantly reduced in WT and A214V mice (5–8% reduction, *p*<0.01) but not in G171V mice (Fig 2F).

Midshaft femur histomorphometric measurements indicated that all genotypes exhibited a significant tail-suspension-induced reduction in periosteal MS/BS (30–50%, *p*<0.05) and periosteal BFR/BS (45–67%, *p*<0.05) compared to genotype-matched controls (S2 Fig and S2 Table), whereas periosteal MAR was significantly reduced by tail suspension only in the A214 mutants. On the endocortical surface, we found significantly lower MAR (38–59%), MS/BS (24–50%), and BFR/BS (49–76%) among tail suspended mice from all three Lrp5 genotypes, compared to their genotyped-matched ground control relatives.

### Lrp5 G171V and A214V HBM mutations impart partial protection against muscle paralysis-induced bone loss

**Fig 3:**
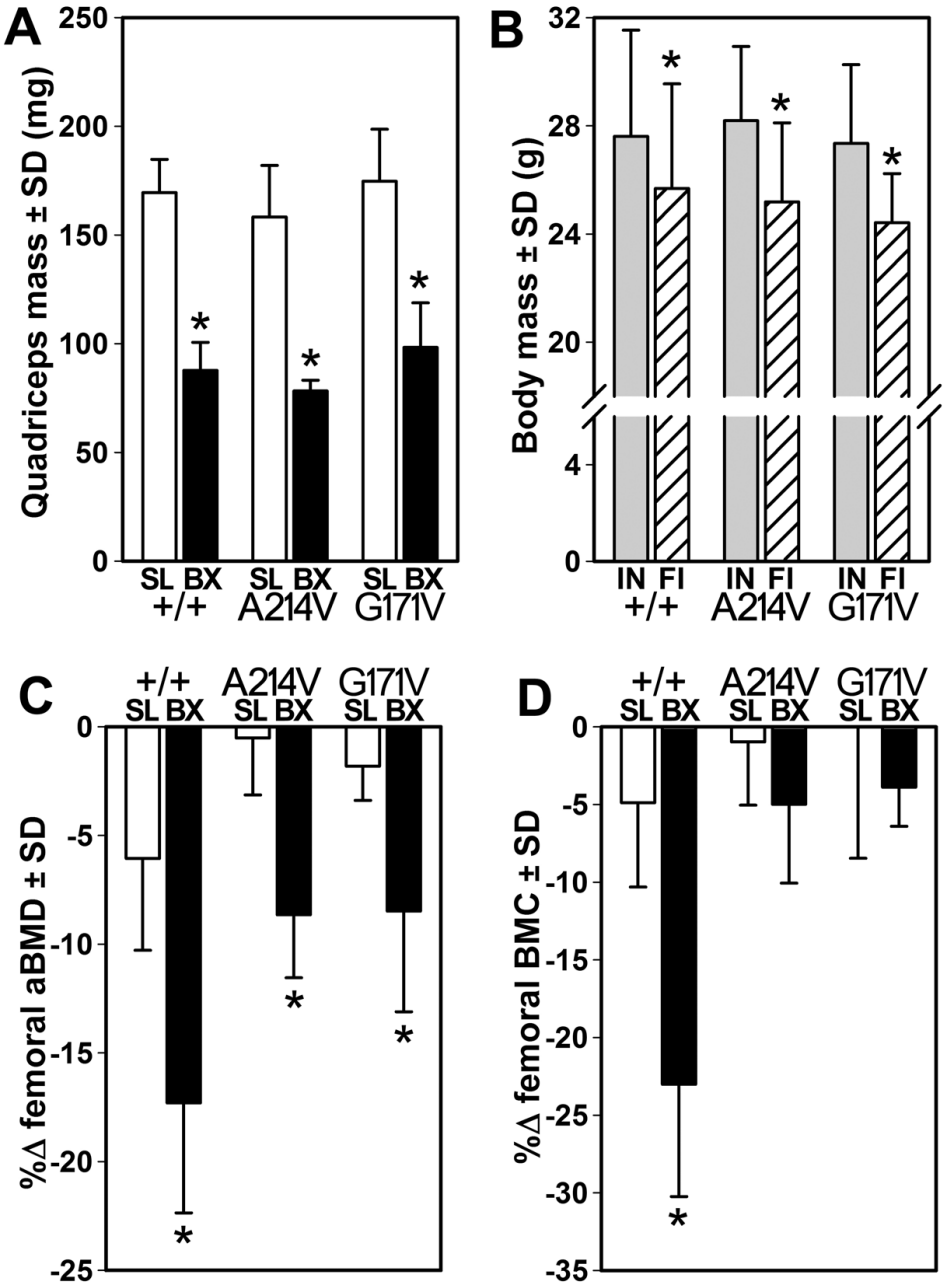
(A) Quadriceps muscle mass was decreased in the botox-injected (BX) limb compared to the saline-injected (SL) limb, among all genotypes. (B) Initial (IN) and final (FI) body weight was similar among all three genotypes. Percent change in femoral bone mineral density (B) and content (C) calculated from DEXA scans collected just prior to the start of the experiment and again at sacrifice. * p<0.05 for botox-treated limb vs. saline-treated limb within genotype. *n* = 8/group, except for WT quadriceps mass (*n* = 7) due to tissue collection error.

To evaluate the bone-wasting effects of a second model of disuse osteoporosis—one involving reduced muscle stimulation due to paralysis—in Lrp5-HBM mice, we treated WT and HBM mice with unilateral hindlimb injection of Botox. To confirm the efficacy of the Botox model, we first evaluated muscle atrophy using the quadriceps muscle weight in each of the three Lrp5 genotypes. Regardless of genotype, Botox treatment of the right hindlimb musculature was associated with a 44–51% decrease (*p*<0.001) in quadriceps weight, compared to the contralateral saline injected quadriceps (Fig 3A). Moreover, the three genotypes lost a statistically equal amount of total body weight over the experimental period (Fig 3B). Next, we evaluated the effects of unilateral Botox-induced muscle paralysis on bone mass and density in Lrp5 WT mice. The femurs of both saline (control) and Botox-injected limbs lost a significant amount of aBMD and BMC, but the Botox-induced losses (17–23% reduction, p<0.001) were significantly greater than those of the saline limb (5–6% reduction, p<0.05; Fig 3C and 3D). In HBM mice, saline-injected limbs exhibited no significant change in aBMD or BMC, except for a mild 2% reduction (p<0.05) in aBMD among G171V mice. The Botox-injected limbs of HBM mice lost both femoral aBMD (9% reduction, *p*<0.01) and BMC (4–5% reduction, *p*<0.05) over the study duration, but the paralysis-induced loss of BMC among the HBM mice was not significantly different from the loss observed in the saline control limbs (Fig 3).

**Fig 4:**
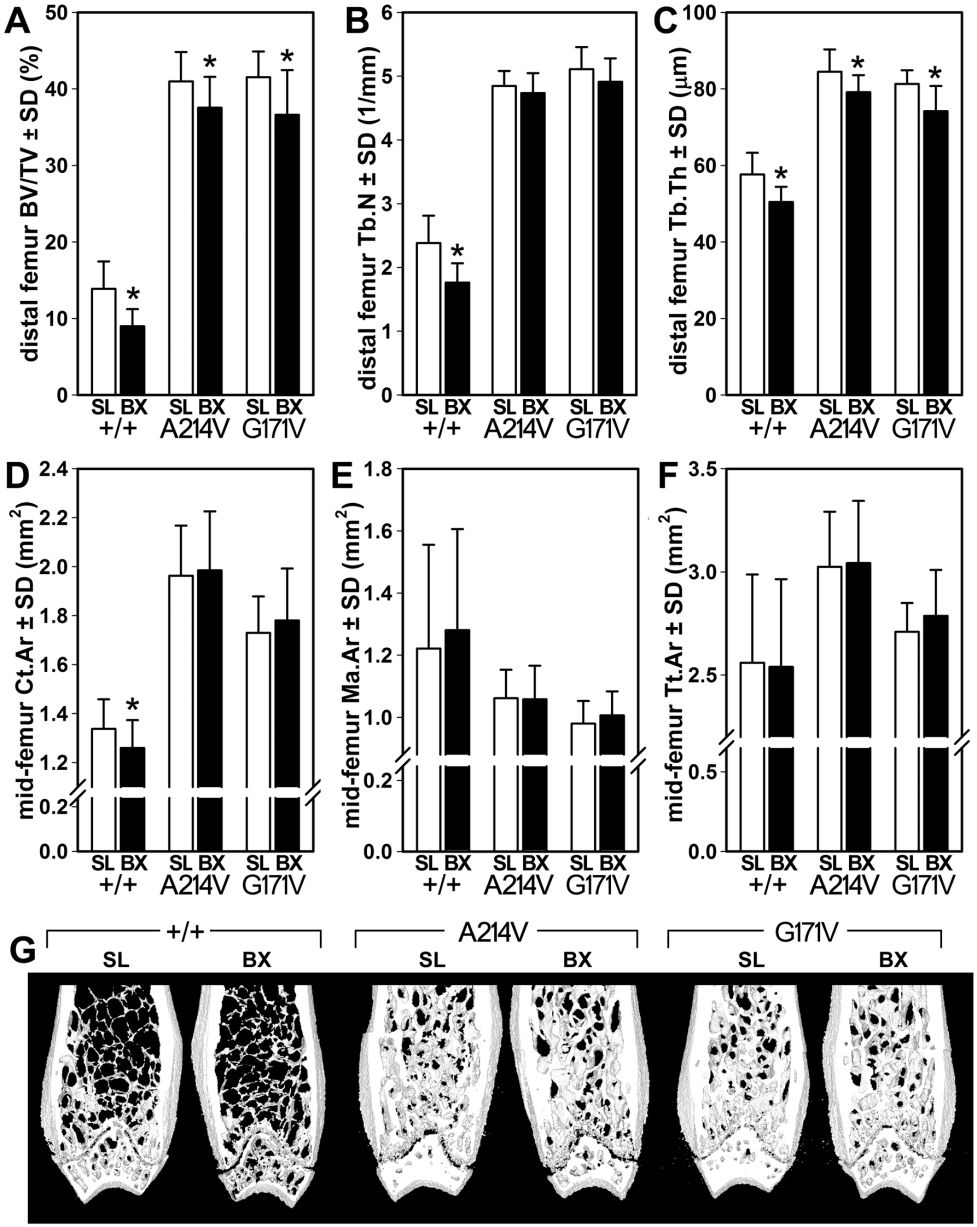
μCT measurements of cancellous and cortical bone properties in the femur from mice that underwent intramuscular injection of saline (SL) into the left lower limb and botox (BX) into the right lower limb. (A) Bone volume fraction, (B) trabecular number, and (C) trabecular thickness were measured in the distal femur metaphysis. (D) Cortical area, (E) medullary area, and (F) total tissue area were measured in the mid-diaphyseal femur. (F) Representative μCT reconstructions of the distal femur from SL- ad BX-treated limbs within each genotype. The anterior and posterior thirds of each reconstruction have been removed digitally to reveal the metaphyseal spongiosa. *p<0.05 for botox-treated limb vs. saline-treated limb within genotype. *n* = 8/group.

Analysis of distal femur cancellous bone by μCT indicated that among WT mice, Botox reduced BV/TV by 35% (*p*<0.01), compared to the saline-injected control limb (Fig 4A). A214V and G171V mice exhibited significant but milder Botox-induced reductions in BV/TV (8% and 12% reduction, respectively, *p*<0.05). Trabecular number was significantly reduced (26% reduction, *p*<0.01) by Botox in WT mice but not in either HBM mutant line (Fig 4B). Trabecular thickness was significantly reduced by Botox treatment in WT mice (13% reduction, *p*<0.01) and in A214V and G171V mice (6% and 9% reduction, respectively, *p*<0.05; Fig 4C). Analysis of the midshaft femur cortex revealed no significant Botox-induced changes in medullary area (Fig 4E) or total tissue area (Fig 4F) for any of the three genotypes. However, cortical area was significantly reduced in WT mice (6% decrease, *p*<0.05) but not in either HBM line (Fig 4D).

Dynamic histomorphometric measurements from the midshaft femur endocortex revealed that the WT mice exhibited a significant Botox-induced reduction in MS/BS (-55%, p<0.01) and BFR/BS (-62%, p<0.05), with no significant change in MAR (S3 Fig, S3 Table). Conversely, both HBM mouse strains exhibited no significant Botox-induced change in any of the endocortical measurements collected. The periosteal surface exhibited few differences between Botox-treated and saline-treated limbs (S3 Table).

### Lrp5 HBM mutations reduce ovariectomy-induced bone loss

**Fig 5:**
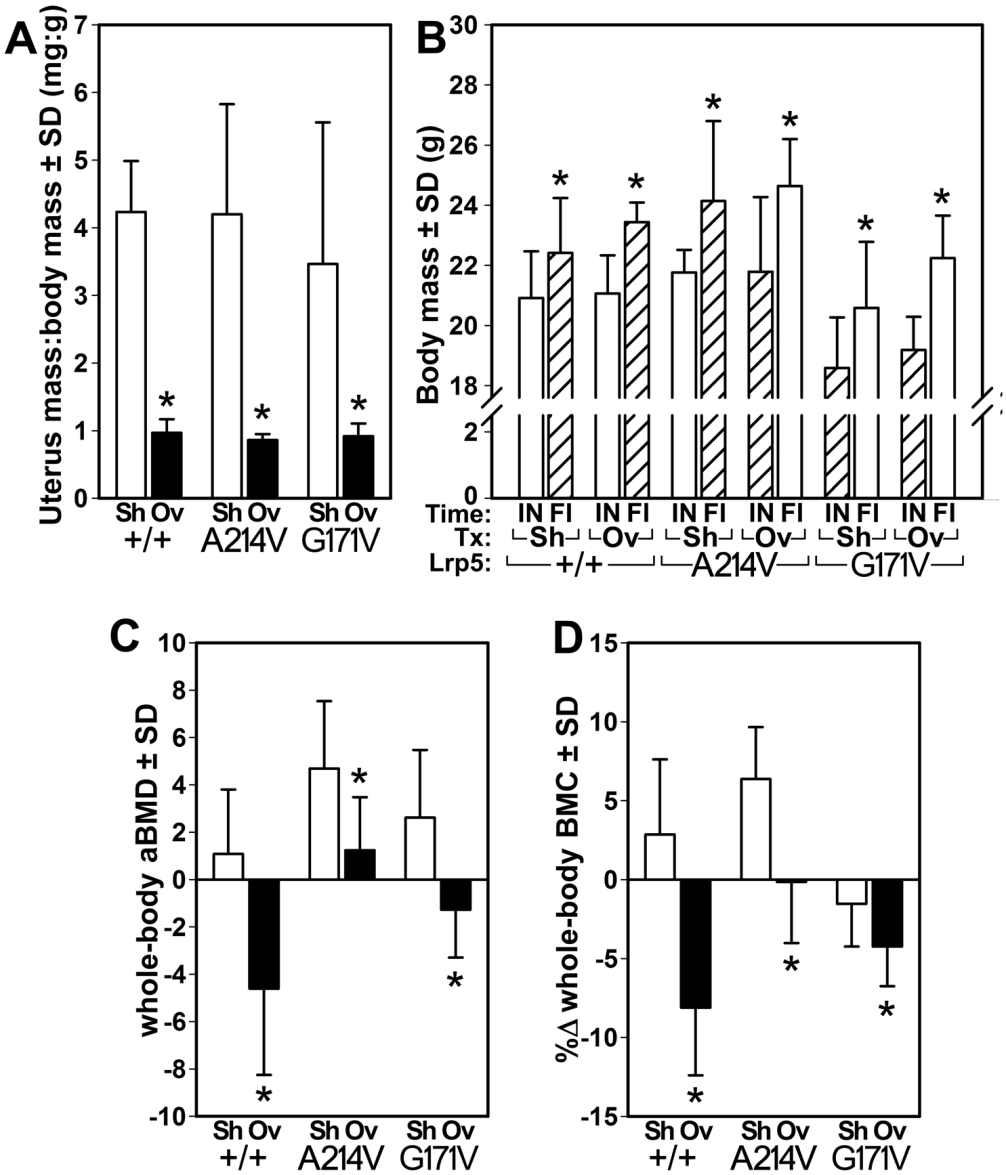
(A) The ratio of uterus wet weight to total body weight was decreased in the mice that underwent ovariectomy surgery (Ov) compared to sham-operated (Sh) littermates, among all genotypes. (B) comparison of initial (IN) and final (FI) body weight was similarly increased among all three genotypes as a result of OVX. Percent change in whole body bone mineral density (B) and content (C) calculated from DEXA scans collected just prior to the start of the experiment and again at sacrifice. * p<0.05 Ovx vs. sham within genotype. *n* = 8/group, except for G171V mice (*n* = 7).

Having found that two different mechanically-based bone wasting stimuli had milder or nullified effects for some bone properties among HBM mice, we sought to learn whether the osteoprotective effects of those mutations were active in nonmechanical bone-wasting contexts. Therefore, we performed ovariectomy (OVX) surgeries in WT, A214V, and G171V mice and measured the skeletal response. To confirm the efficacy of the ovariectomy (OVX) model in our experiments, we first evaluated changes in uterus weight in ovariectomized and sham-operated mice. Regardless of genotype, OVX was associated with a 74–80% decrease (*p*<0.01), in the uterus weight:body weight ratio, compared to genotype-matched sham-operated animals (Fig 5A), indicating a successful reduction in estrogen levels via surgical intervention (ovary removal). Moreover, the three genotypes gained a similar amount of total body weight over the experimental period (Fig 5B). Next, we evaluated the effects of ovariectomy on whole-body bone mass and density. WT sham-operated mice exhibited no significant change in aBMD or BMC over the study duration (Fig 5C and 5D). OVX in WT mice resulted in a significant loss of whole body aBMD (-5%; *p*<0.01) and BMC (-8%; *p*<0.05). Both A214V and G171V mice subjected to sham surgery gained a significant amount of whole body aBMD (+5% and +3%, respectively, *p*<0.05), though only A214V sham-operated gained BMC (+6%, *p*<0.001). OVXed A214V and G171V mice exhibited no significant changes in whole body aBMD or BMC over the duration (change not significantly different from zero), with the exception of the G171V mice, which lost -4% aBMD (*p*<0.01).

**Fig 6:**
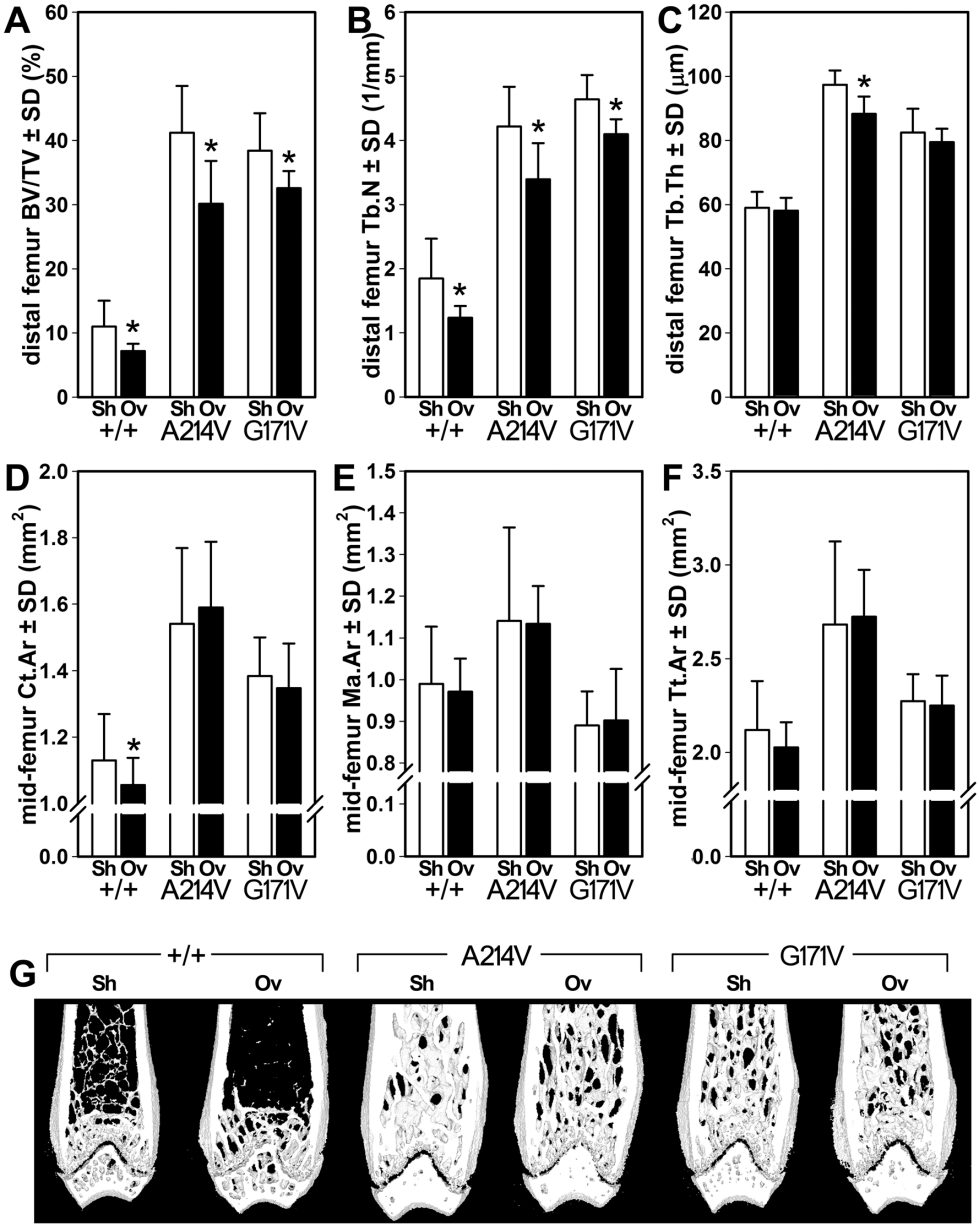
μCT measurements of cancellous and cortical bone properties in the femur from mice that underwent ovariectomy surgery (Ov) or sham surgery (Sh). (A) Bone volume fraction, (B) trabecular number, and (C) trabecular thickness were measured in the distal femur metaphysis. (D) Cortical area, (E) medullary area, and (F) total tissue area were measured in the mid-diaphyseal femur. (F) Representative μCT reconstructions of the distal femur from OVX- and sham-operated mice within each genotype. The anterior and posterior thirds of each reconstruction have been removed digitally to reveal the metaphyseal spongiosa. *p<0.05 for OVX vs. sham within genotype. *n* = 8/group, except for G171V mice (*n* = 7).

In the distal femur metaphysis, OVXed WT mice exhibited significantly lower BV/TV (35% reduction, *p*<0.05) compared to sham (Fig 6A). OVX resulted in a -27% and -15% lower BV/TV (*p*<0.05) in A214V and G171V mice, respectively. Trabecular number was significantly reduced by OVX in all three genotypes (33, 19, and 12% reductions in WT, A214V, and G171V, respectively; *p*<0.05, Fig 6B). Trabecular thickness was reduced by OVX only in the A214V mice (Fig 6C). Cancellous parameters in the fifth lumbar vertebral body (L5) revealed that the G171V mutation was protective against OVX-induced trabecular bone loss (S4 Fig and S4 Table). The OVX procedure in WT mice resulted in significantly reduced BV/TV, Tb.N, and Tb.Sp, whereas the same outcomes were not altered in OVXed G171V mice. A214V mice exhibited a significant OVX-induced loss of BV/TV and Tb.N. Midshaft femur cortical area was significantly reduced by OVX in WT (7% reduction, *p*<0.05) but not A214V or G171V mice (Fig 6D). Medullary area and total tissue area were statistically unchanged by OVX in all three groups (Fig 6E and 6F), though the OVX-induced reduction in total tissue area among WT mice approached statistical significance (*p* = 0.06).

Evaluation of midshaft femur histomorphometric indices from the periosteal and endocortical surfaces yielded no significant differences within any Lrp5 genotype as a result of OVX (S5 Fig and S5 Table).

## Discussion

We initiated a set of osteocatabolic experiments to determine if the LRP5 high bone mass (HBM) missense mutations, A214V and G171V, have protective effects on the bone wasting response to mechanical disuse in the growing and mature mouse skeleton. We subjected LRP5 HBM knock-in mice to two different models of disuse osteoporosis—tail suspension and muscle paralysis. As a control experiment, we subjected HBM mice to a non-mechanical bone wasting stimulus—ovariectomy (OVX). In both disuse models, we observed differences in our endpoints between bones from the control animal/leg and the mechanically deprived animal/leg, indicating that disuse affects the skeleton to some degree in HBM mice, albeit to a lesser extent than in WT mice. The question of whether the HBM alleles can prevent disuse induced bone loss *per se* (as opposed to the separate question of whether disuse affects bone accumulation), can only be addressed using the DEXA data, where we have measurements of lower limb bone mass prior to disuse, and again after 3 wks of disuse. Those measurements indicate that the HBM alleles are able to prevent, or nearly prevent, the disuse-induced bone loss seen in WT animals. For example, tail suspension treatment resulted in a significant ~4% decrease in femoral BMD among WT mice, whereas HBM mice exhibited no change, or a significant increase, in femur BMC as a result of tail suspension. A similar picture emerges for the Botox experiment: Botox treatment resulted in a significant ~23% decrease in femur BMC among WT mice, whereas the same treatment applied to HBM mice resulted in a modest 4–5% decrease in femur BMC. While that decrease in bone properties among HBM mice is significant, it is several-fold less than that observed for WT mice. Taken together, these data suggest that the HBM alleles confer significant, nearly complete, protection from bone loss associated with disuse.

The μCT data from the distal femur and lumbar spine indicate significant and roughly equal disparities between control bones and disuse-treated bones, suggesting that trabecular bone is susceptible to disuse effects whether HBM or WT alleles are present. While these results may appear to contradict the serial BMC data discussed above, the μCT parameters are single endpoints and are unable to reflect changes in the trabecular parameters over time (i.e. whether the trabecular bone was maintained or reduced in the suspended mice). Assessment of actual trabecular bone loss, rather than a failure to gain bone, would require an additional baseline control group for each genotype that was sacrificed on the day that disuse was induced. Thus it is unclear whether disuse induced significant trabecular bone loss in the HBM mice, or whether, like the DEXA data highlight for the whole leg, those mice failed to continually increase their trabecular bone during the experimental period (as might have occurred in the saline-treated limb). From a functional perspective, the trabecular bone parameters in HBM suspended mice were consistently greater than those of WT control mice, indicating that the HBM mutants were, at the very least, partially protected from suspension-induced osteopenia during the study and were able to still maintain a HBM phenotype.

Using a loss-of-function mouse model of Lrp5 signaling, we previously reported that mechanical (ulnar loading) but not hormonal (intermittent PTH) stimuli are compromised by a lack of Lrp5 expression [40]. Likewise, preliminary experiments in an Lrp5 gain-of-function transgenic mouse model (^3.6kb^ColIa1::Lrp5G171V) show that mechanical (sciatic neurectomy) but not hormonal (OVX) challenges are associated with preserved bone mass when G171V is overexpressed [41]. To assess whether the bone-sparing effects of Lrp5 HBM alleles are manifest in catabolic conditions other than mechanical disuse in our models, we exposed the HBM and WT mice to the hormonal challenge of estrogen deprivation via OVX. We found that whereas the WT mice exhibited significant losses in whole body aBMD and BMC as a result of OVX, the HBM mice were largely protected from those losses. Similar to the disuse studies, the OVX mice had less trabecular bone in the femur and spine than the sham-surgery mice, regardless of Lrp5 genotype. An exception to this trend was among the G171V mice, which exhibited protection from OVX-induced trabecular bone effects in most of the μCT measurements made. Again, it is unclear whether the trabecular bone in the A214V mice was protected from loss, or if failure to gain bone during the experimental period can account for the discrepancies. In either case, the bone-protective effects of Lrp5 HBM alleles are not restricted to mechanical challenges, but also appear to be active in estrogen-deficient environments.

We have previously found that both HBM mutations are protected from the bone loss induced by Sost overexpression. It has also been reported that tail-suspension induced bone loss is mediated by an increased expression of Sost, and that genetic deletion of Sost or antibody-mediated inhibition of sclerostin prevents suspension-induced bone loss [18, 42]. We report here in our two disuse experiments that the HBM mutations protect against disuse-induced cortical bone loss, but are unable to maintain normal (accelerated) bone growth associated with the HBM alleles. These observations present a potential conundrum: Sost^-/-^ mice are completely immune to disuse effects in bone, yet the sclerostin-resistant Lrp5 HBM mice only maintain bone during disuse.

Several possibilities can be invoked to explain this discrepancy. First, while Sost is required to mediate disuse-induced bone loss, another signaling component may also be required, such as a corresponding decrease in Wnts. In this scenario, disuse in WT mice results in reduced Wnt and increased Sost (which might then fully suppress Wnt signaling), while disuse in HBM results in similarly reduced Wnt expression without being affected by the increased Sost (thus allowing some Wnt signaling that allows maintenance of bone mass). Another possible explanation is that disuse-induced Sost expression may be increased to a higher extent than that caused by ^8kb^Dmp1::Sost overexpression (i.e., ^8kb^Dmp1::Sost mouse is not a phenocopy of a tail-suspended mouse), thereby allowing sclerostin to suppress Lrp5 signaling to a fuller extent than is seen in the overexpression model. Alternatively, the effects might be related to sclerostin’s other targets, including Lrp6 and Lrp4. In the Sost^-/-^ mice, both Lrp5 and Lrp6 would be expected to experience Wnt signaling uninhibited by sclerostin, whereas in our Lrp5 HBM knock-ins, Lrp6 would be expected to experience the full effects of sclerostin-mediated inhibition of that receptor. Further, it is unclear how these mutations affect the expression of or interaction with a recently identified cofactor—Lrp4—for sclerostin’s inhibitory action on Lrp5/6 signaling [43–45].

While the disuse experiments we report support a consistent theme of reduced bone loss in HBM mice, there are some discrepancies between the two models. First, it should be noted that the Botox injections were performed on 16-wk-old mice and the hindlimb suspension experiments were performed on a 10-wk-old mice, so comparison between these two models is not truly a direct one due to the confounding effects of growth in the hindlimb suspension studies. Second, we did detect a small systemic effect (i.e., in the saline-injected left leg) induced by local Botox injections in the right leg musculature. Comparison of the DEXA-derived changes in bone mass in the ground control mice from the tail suspension experiment, with DEXA-derived changes in bone mass in the saline-treated leg of the Botox animals reveals a noticeable suppression of bone gain in the presence of contralateral Botox exposure. Similar observations have been reported previously [46], but it is unclear whether this effect is fueled by Botox compound entering the systemic circulation and modestly suppressing muscle function elsewhere in the body, or perhaps by reducing overall mobility (normal functional loading in the control leg) because of the contralateral paralysis. The use of two disuse models has a clear advantage in overcoming the experimental limitations of either model, and in our case, has added considerable strength to the observation that the HBM alleles have a protective effect on disuse-induced bone loss.

Previous reports on ovariectomy in the transgenic HBM mouse, ^3.6kb^Col1a1::Lrp5^G171V^, indicated that the mice lost bone at the same rate as WT mice, suggesting that they were not protected from bone loss due to sex hormone deficiency [41]. However, the same mouse model was protected from sciatic neurectomy induced bone loss [47]. We found that the HBM knock-in mice were much less affected by OVX than their WT controls. Although our disuse experiments were conducted on mice of differing age (16 wks for botox experiments vs 10 wks for tail suspension [and OVX]), the differences in response to challenge in out mice might be due to genetic engineering differences between the models (e.g., knock-in strategy versus the transgenic approach). Our knock-in model expresses Lrp5 according to the endogenous spatial–temporal profile and is not limited to or enhanced by expression of the ^3.6kb^ColIa1 promoter. We found that although our HBM mice were unable to accrue bone (compare to shams), they did not significantly lose aBMD or BMC over time, with the exception of one measurement. These results highlight the potential therapeutic value of targeting this pathway pharmacologically in order to prevent menopause induced bone loss.

In conclusion, these results give support to the hypothesis that the Lrp5 HBM mutations protect against bone loss in the presence of bone wasting stimuli. We found that the disuse stimuli induced significant bone loss in WT mice, but Lrp5 A214V and G171V were partially or fully protected from the bone loss that normally results from disuse. Trabecular bone parameters among HBM mice were significantly affected by disuse in both models, but these data are consistent with DEXA data showing a failure to continue growing in HBM mice, rather than a loss of pre-existing bone. Surprisingly, ovariectomy in Lrp5 HBM mice resulted in similar protection from bone catabolism. The Lrp5 HBM alleles offer significant protection from the catabolic effects of disuse and estrogen deficiency, and consequently, present a potential mechanism to target pharmacologically to protect against various bone-wasting stimuli.

## Supporting Information

S1 Fig Representative μCT reconstructions of the fifth lumbar vertebra from GC and TS mice within each genotype. The anterior and posterior thirds of each reconstruction have been removed digitally to reveal the vertebral body spongiosa. See S1 Table for fifth lumbar vertebral cancellous measurements.
(TIF)

S2 Fig Representative fluorochrome-labeled midshaft femur sections from ground control (GC) and tail suspended (TS) mice. Macro-scale images of the entire cross section are shown to the left, and close-up panels of the areas indicated by the white boxes are shown at higher magnification to the right. Although several fluorochromes were injected to monitor bone formation, data were collected using the pretreatment label (tetracycline, indicated by gold arrow) and the calcein label (green arrow), which was given close to the sacrifice date. The tetracycline label is not as strong as the other labels and is difficult to visualize in many of the photomicrographs.
(TIF)

S3 Fig Representative fluorochrome-labeled midshaft femur sections from the saline-treated limb and the botox-treated limb. Macro-scale images of the entire cross section are shown to the left, and close-up panels of the areas indicated by the white boxes are shown at higher magnification to the right. Although several fluorochromes were injected to monitor bone formation, data were collected using the pretreatment label (tetracycline, indicated by gold arrow) and the calcein label (green arrow), which was given close to the sacrifice date. The tetracycline label is not as strong as the other labels and is difficult to visualize in many of the photomicrographs.
(TIF)

S4 Fig Representative μCT reconstructions of the fifth lumbar vertebra from OVX- and sham-operated mice within each genotype. The anterior and posterior thirds of each reconstruction have been removed digitally to reveal the vertebral body spongiosa. See S4 Table for fifth lumbar vertebral cancellous measurements.
(TIF)

S5 Fig Representative fluorochrome-labeled midshaft femur sections from OVX- and sham-operated mice. Macro-scale images of the entire cross section are shown to the left, and close-up panels of the areas indicated by the white boxes are shown at higher magnification to the right. Although several fluorochromes were injected to monitor bone formation, data were collected using the pretreatment label (tetracycline, indicated by gold arrow) and the alizarin label (red arrow), which was given close to the sacrifice date. The tetracycline label is not as strong as the other labels and is difficult to visualize in many of the photomicrographs.
(TIF)

S1 Table Summary of L5 trabecular parameters in the tail suspension study. (XLSX)

S2 Table Summary of femoral midshaft dynamic histomorphometric parameters in the tail suspension study. (XLSX)

S3 Table Summary of femoral midshaft dynamic histomorphometric parameters in the botox study. (XLSX)

S4 Table Summary of L5 trabecular parameters in the ovariectomy study. (XLSX)

S5 Table Summary of femoral midshaft dynamic histomorphometric parameters in the ovariectomy study. (XLSX)
