# Supplementary figures and images for: Missense Mutations in LRP5 Associated with High Bone Mass Protect the Mouse Skeleton from Disuse- and Ovariectomy-Induced Osteopenia
Source: PLoS One. 2015 Nov 10;10(11):e0140775. doi: 10.1371/journal.pone.0140775 (PMC4640505; doi:10.1371/journal.pone.0140775)

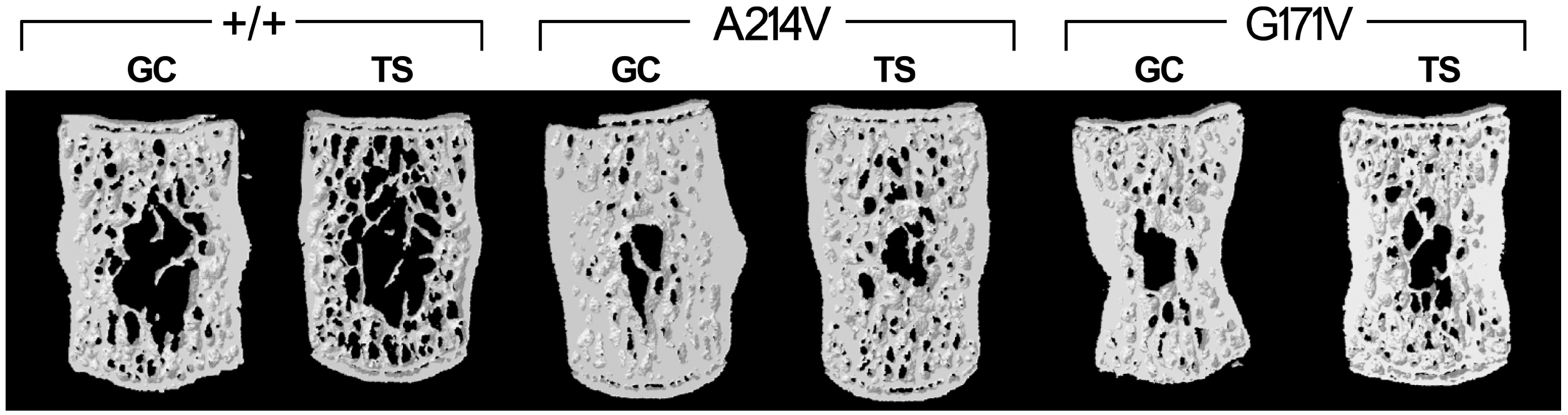

Supplement: S1 Fig — The anterior and posterior thirds of each reconstruction have been removed digitally to reveal the vertebral body spongiosa. See S1 Table for fifth lumbar vertebral cancellous measurements. (TIF) [file pone.0140775.s001.tif]

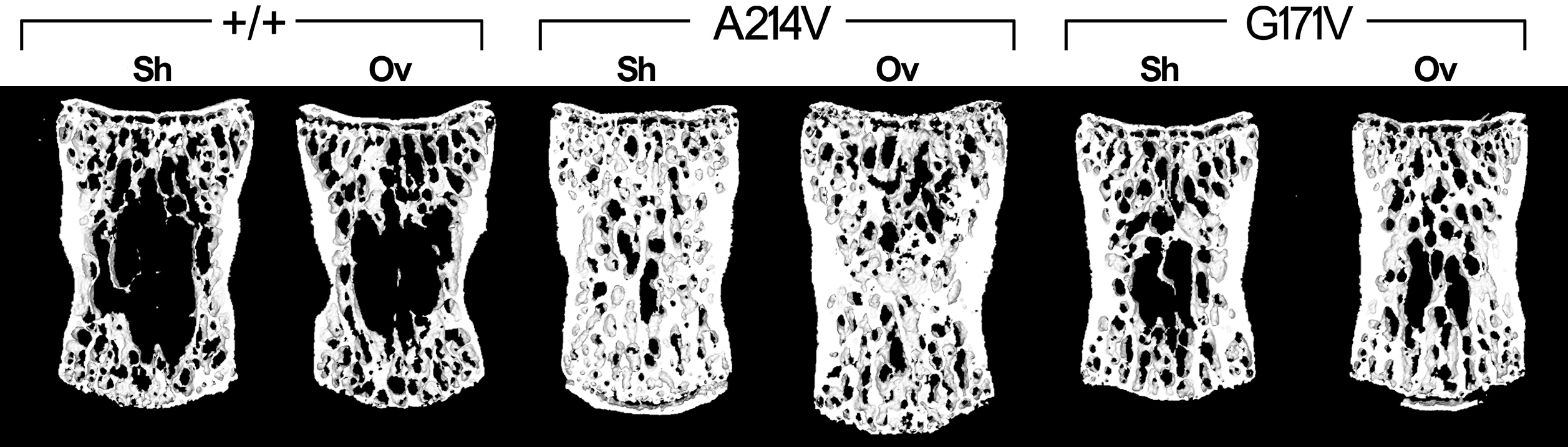

Supplement: S4 Fig — The anterior and posterior thirds of each reconstruction have been removed digitally to reveal the vertebral body spongiosa. See S4 Table for fifth lumbar vertebral cancellous measurements. (TIF) [file pone.0140775.s004.tif]
